# Topic model-based mass spectrometric data analysis in cancer biomarker discovery studies

**DOI:** 10.1186/s12864-016-2796-x

**Published:** 2016-08-18

**Authors:** Minkun Wang, Tsung-Heng Tsai, Cristina Di Poto, Alessia Ferrarini, Guoqiang Yu, Habtom W. Ressom

**Affiliations:** 1 Department of Oncology, Georgetown University, 4000 Reservoir Rd NW, Washington D.C., USA; 2 Department of Electrical and Computer Engineering, Virginia Tech, 900 N Glebe Rd, Arlington, VA USA

**Keywords:** Bayesian inference, Topic model, Purification, LC-MS, GC-MS, Extracted ion chromatogram, Metabolomics, Proteomics, Biomarker discovery

## Abstract

**Background:**

A fundamental challenge in quantitation of biomolecules for cancer biomarker discovery is owing to the heterogeneous nature of human biospecimens. Although this issue has been a subject of discussion in cancer genomic studies, it has not yet been rigorously investigated in mass spectrometry based proteomic and metabolomic studies. Purification of mass spectometric data is highly desired prior to subsequent analysis, e.g., quantitative comparison of the abundance of biomolecules in biological samples.

**Methods:**

We investigated topic models to computationally analyze mass spectrometric data considering both integrated peak intensities and scan-level features, i.e., extracted ion chromatograms (EICs). Probabilistic generative models enable flexible representation in data structure and infer sample-specific pure resources. Scan-level modeling helps alleviate information loss during data preprocessing. We evaluated the capability of the proposed models in capturing mixture proportions of contaminants and cancer profiles on LC-MS based serum proteomic and GC-MS based tissue metabolomic datasets acquired from patients with hepatocellular carcinoma (HCC) and liver cirrhosis as well as synthetic data we generated based on the serum proteomic data.

**Results:**

The results we obtained by analysis of the synthetic data demonstrated that both intensity-level and scan-level purification models can accurately infer the mixture proportions and the underlying true cancerous sources with small average error ratios (<7 *%*) between estimation and ground truth. By applying the topic model-based purification to mass spectrometric data, we found more proteins and metabolites with significant changes between HCC cases and cirrhotic controls. Candidate biomarkers selected after purification yielded biologically meaningful pathway analysis results and improved disease discrimination power in terms of the area under ROC curve compared to the results found prior to purification.

**Conclusions:**

We investigated topic model-based inference methods to computationally address the heterogeneity issue in samples analyzed by LC/GC-MS. We observed that incorporation of scan-level features have the potential to lead to more accurate purification results by alleviating the loss in information as a result of integrating peaks. We believe cancer biomarker discovery studies that use mass spectrometric analysis of human biospecimens can greatly benefit from topic model-based purification of the data prior to statistical and pathway analyses.

## Background

Identification of disease-related alterations in molecular and cellular mechanisms may reveal useful disease biomarkers. Discovery of clinically relevant biomarkers has potentially far reaching implications for disease management and patient treatment [1–4]. High-throughput omic technologies have facilitated the search for changes in the levels of various biomolecules (proteins, glycoproteins, metabolites, lipids, etc.) in biological samples [5, 6]. In particular, liquid (or gas) chromatography coupled with mass spectrometry (LC/GC-MS) has become an essential tool for profiling biomolecules in a variety of large-scale omic studies. Briefly, biomolecules are separated, fragmented, ionized and detected in LC/GC-MS instruments. Abundances of ions with various retention time and mass values are recorded for downstream data processing.

While the capability of high-throughput technology to yield comprehensive profiling and quantification offers a unique advantage in biomedical research, the heterogeneous nature of the biological samples poses a fundamental challenge in data analysis and interpretation. Specimens, such as tumor tissues and human blood, are typically mixtures of cells with distinct states and types, and usually only part of the constituent cell populations is relevant to the biological question of interest [7, 8]. In some cancer studies, heterogeneity is also observed within the malignant cell population, where multiple cancerous subtypes co-exist [9]. Ideally in a biomarker discovery study, one would perform between-group (cancer versus related disease, cancer versus healthy samples) differential expression analysis for type-specific constituents in samples [10]. However, biospecimens collected from patients usually exhibit some degree of heterogeneity. Moreover, the proportion of cancerous, other disease-related, and healthy components varies across individual samples pre-selected using pathological estimates. Therefore, the biomolecular measurements in expression profiles are attributed to multiple sites of origins with various mixture proportions. The cancerous profiles of interest are typically contaminated by other components, leading to unreliable results in differential analyses. Purification of samples is hence highly desired to remove the effects of heterogeneity.

Experimental methods for cleaning samples and isolating type-specific constituents are costly and time-consuming. Computational purification methods offer an attractive alternative that is inexpensive and efficient to implement, and can be applied to data already generated without any modifications on experimental procedures. Multiple approaches have been developed to deconvolute gene expression profiles in the past years, varying from linear regression based models [11, 12] to generative probabilistic models [13, 14]. Among these approaches, topic model based methods, e.g., ISOLATE [15] and ISOpure [8], showed promising performance in estimating the proportion of mixtures and inferring sample-specific purified profiles in heterogeneous genomic data. However, to the best of our knowledge, in omic studies involving quantitative analysis of proteins or metabolites, no such purification approaches have been applied to deal with the sample heterogeneity issue. With the increasing volume of these data generated by LC/GC-MS, it is necessary to implement the purification of data before downstream differential analyses. In this research, we first apply ISOpure, a topic model based purification approach to both synthetic and experimental data acquired from human sera and liver tissues by LC-MS and GC-MS, respectively. The purpose of this investigation is to test if sample heterogeneity issue in various biomolecular expression profiles can be addressed by adjusting ion intensities through topic models as in genomic studies. Also, we investigate the use of scan-level features, i.e. extracted ion chromatograms (EICs) instead of integrated peak intensities, to alleviate the information loss during the LC/GC-MS data preprocessing.

## Methods

In this section, we introduce topic model-based intensity-level and scan-level purification methods. Assumptions and strategies in the topic models are elaborated. Mass spectrometric datasets from cancer biomarker discovery studies are described.

### Intensity-level purification model

The LC/GC-MS instruments provide ion intensity values by counting the ions detected at specific *m*/*z* and retention time points. Due to the existence of heterogeneity, multiple constituents in the sample contribute to the observed expression profile. Therefore, we can model the expression profile of a heterogeneous sample ***t*** as a weighted mixture of expression profiles of multiple sources, including a cancerous origin ***γ*** and non-cancerous contaminants ***β***. The expression distribution for every biomolecule in each of the sources plays a role as a “topic” contributing to the mixed expression profile. Basically, each ion in the observed profile is associated with a specific topic, i.e. a multinomial distribution of ion counts over biomolecules, determined by the corresponding source profile. In this model, expression profiles refer to integrated peak intensities.

The purification procedure can be realized through a set of topic models, which are generative probabilistic models typically applied to text corpora mining. Specifically, each expression profile is characterized by a probability distribution across topics. Topics are probability distributions across biomolecules. These distributions can be inferred based on the analysis of a collection of expression profiles through topic models. These hierarchical Bayesian models are variants of latent Dirichlet allocation (LDA) [16], a topic model that can 1) infer the posterior probability of topics given observed profiles, and 2) estimate the parameters that generate the latent mixture proportion and topic panel. These topic models have been adapted and applied to gene expression profiles in genomic studies [8, 15].

We use a modified topic model to purify the molecular expression profiles in cancer. Basically, three assumptions are made in developing the model. First, the source contaminants in each expression profile {***t***_*d*_}_*d*=1,⋯,*D*_ are coming from the control group {***β***_*m*_}_*m*=1,⋯,*M*_ (i.e., healthy, non-cancerous profiles, etc.). It is commonly observed that the cancerous tissues are surrounded by adjacent non-cancerous tissues, which are typically used as controls in differential expression analysis. Second, the corresponding cancerous origins {***γ***_*d*_}_*d*=1,⋯,*D*_ share an average cancer profile ***γ***^***′***^. Individual cancerous profile can be treated as a noisy version of the average cancer profile. Third, the average cancer profile ***γ***^***′***^ has similar patterns as non-cancerous profiles {***β***}, except for some sites (biomolecules) which are differentially expressed between case and control groups. Thus, the cancerous profile can be treated as a similar non-cancerous profile with several sites altered.

The complete likelihood function in (1) describes how the profiles {***t***_*d*_}_(*d*=1,…,*D*)_ are generated. Specifically, we have two observable variables indicating *D* expression profiles in case group: $\{\boldsymbol {t}_{d}\}_{d=1,\cdots,~D},~\boldsymbol {t}_{d}\in \mathbb {R}^{N}$, and *M* non-cancerous profiles in control group: $\{\boldsymbol {\beta }_{m}\}_{m=1,\cdots,M},~\boldsymbol {\beta }_{m}\in \mathbb {R}^{L}$. In our analysis, we normalize all profiles to have identical total ion counts of *N* and consider *L* biomolecules that are consistently detected in all the samples. For convenience, we represent the normalized profiles in two ways. Each heterogeneous cancer profile ***t***_*d*_ is represented via *N* ions, with *t*_*d,n*_={1,2,⋯,*L*} denoting the biomolecule corresponding to the *n*^th^ ion. Each non-cancerous profile ***β***_*m*_ is represented via *L* biomolecules, with *β*_*m,l*_ denoting the ion counts of the *l*^th^ biomolecule, and $\sum _{l=1}^{L}\beta _{m,l} = N$. The second expression can be further normalized by *N* to give a representation of multinomial distribution as a topic.

1 $$  \begin{aligned} \mathcal{L}(\boldsymbol{t},\boldsymbol{z},\boldsymbol{\theta},&\boldsymbol{\gamma},\boldsymbol{\gamma'}|\alpha,\boldsymbol{\beta},\boldsymbol{\eta},\kappa, \kappa')\\ &= p\left(\boldsymbol{\gamma'}|\boldsymbol{\beta},\boldsymbol{\eta},\kappa'\right)\cdot \prod_{d=1}^{D} p\left(\boldsymbol{\theta}_{d}|\alpha\right)\cdot p\left(\boldsymbol{\gamma}_{d}|\boldsymbol{\gamma'},\kappa_{d}\right) \\ & \quad\times \prod_{n=1}^{N}\left[p\left(z_{d,n}|\boldsymbol{\theta}_{d}\right)\cdot p\left(t_{d,n}|z_{d,n}, \boldsymbol{\theta}_{d}, \boldsymbol{\beta},\boldsymbol{\gamma}_{d}\right)\right] \end{aligned}  $$

The model also incorporates the following latent variables: the average cancer profile $\boldsymbol {\gamma }'\in \mathbb {R}^{L}$, sample-specific pure cancer profiles $\{\boldsymbol {\gamma }_{d}\}_{d =1,\cdots,D}, ~\boldsymbol {\gamma }_{d} \in \mathbb {R}^{L}$, sample-specific mixture proportions of topics $\{\boldsymbol {\theta }_{d}\}_{d=1,\cdots,D},\boldsymbol {\theta }_{d}\in \mathbb {R}^{M+1}$, and sample-specific topic indicators $\{\boldsymbol {z}_{d}\}_{d=1,\cdots,D}, \boldsymbol {z}_{d}\in \mathbb {R}^{N},~ z_{d,n} = \{1,\cdots,M,M+1\}$. Their relationships with observations and parameters are given as below.

2 $$ p\left(\boldsymbol{\theta}_{d}|\boldsymbol{\alpha}\right) = \text{Dirichlet}\left(\boldsymbol{\theta}_{d}|\boldsymbol{\alpha},1\right)~~  $$

3 $$ p\left(\boldsymbol{\gamma'}|\boldsymbol{\beta},\eta, \kappa'\right) = \text{Dirichlet}\left(\boldsymbol{\gamma}'|\boldsymbol{\eta}^{T}\boldsymbol{\beta},\kappa'\right)~~~~  $$

4 $$ p\left(\boldsymbol{\gamma}_{d}|\boldsymbol{\gamma}',\kappa_{d}\right) = \text{Dirichlet}\left(\boldsymbol{\gamma}_{d}|\boldsymbol{\gamma}',\kappa_{d}\right)~~~~~  $$

5 $$ p\left(z_{d,n}|\boldsymbol{\theta}_{d}\right) = \text{Multinomial}\left(z_{d,n}|\boldsymbol{\theta}_{d}\right)  $$

6 $$ p\left(t_{d,n}|z_{d,n}\le M, \boldsymbol{\theta}_{d}, \boldsymbol{\beta},\boldsymbol{\gamma}_{d}\right) = \text{Multinomial}\left(t_{d,n}|\boldsymbol{\beta}_{z_{d,n}}\right)  $$

7 $$  p\left(t_{d,n}|z_{d,n}= M+1, \boldsymbol{\theta}_{d}, \boldsymbol{\beta},\boldsymbol{\gamma}_{d}\right) = \text{Multinomial}\left(t_{d,n}|\boldsymbol{\gamma}_{d}\right)  $$

**Fig. 1:**
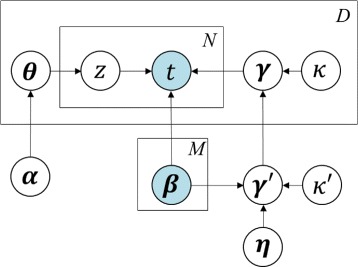
Graphical representation of the generative probabilistic model. Hyperparameters ***η***, *κ* ^′^ together with sources of contaminants {***β***_*m*_} determine an average cancer profile ***γ*** ^***′***^. Each of the *D* profiles is associated with a mixture proportion ***θ***_*d*_ (regularized by hyperparameter *α*) and a topic panel consisting of {***β***_*m*_} and *γ* ^′^ (generated from the average cancer profile). Each of the *N* ions in a profile *t*_*n,d*_ is sampled from a topic indicated by *z*_*n,d*_

The average cancer profile ***γ***^′^ is sampled from a Dirichlet distribution parameterized by a weighted mixture of non-cancerous profiles. Each pure cancer profile ***γ***_*d*_ together with *M* contaminants {***β***_*m*_} forms a sample-specific topic panel. The mixture proportion of topics determines *z*_*d,n*_, indicating which source (i.e., *γ*_*d*_ or {***β***_*m*_}) each ion originates from. We infer the latent variables ***γ***^′^,{***γ***_*d*_}_*d*=1,⋯,*D*_,{***θ***_*d*_}_*d*=1,⋯,*D*_, and estimate the parameters using the two-step learning approach developed based on variational EM algorithm (ISOpure package [8], version 1.4). The graphical model representing the above topic model is shown in Fig. 1. This three-level model allows a single profile to be associated with multiple topics (i.e., cancerous and non-cancerous origins). Such property of the LDA-framed models enable more flexible representation in data structure than that by other unigram models or mixture of unigrams [16]. Also in contrast to linear regression models, these methods use a multinomial noise model that is a better fit to noise measurement in biomolecular expression data [13].

### Scan-level purification model

Here, we extend the topic model to utilize the scan-level measurements instead of the integrated peak intensities. During LC/GC-MS data preprocessing, ion intensity is obtained by integrating the scan-level measurements of a detected chromatographic peakl within a specified retention time (RT) interval. This integration or truncation, however, inevitably brings in variances which interfere with original sample heterogeneity. Therefore, we propose to investigate LC/GC-MS data purification with scan-level measurements based on extracted ion chromatogram (EIC), which preserves scan-level peak shape information. We hypothesize that purification at the scan level leads to more accurate results and offers the opportunity to extend the model to characterize both ion abundance and peak shape.

**Fig. 2:**
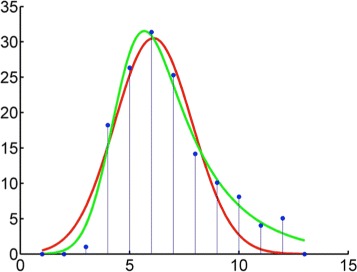
Extracted ion chromatography and peak shape function. Example of Gaussian (*red*) and exponentially modified Gaussian (*green*) peak shapes fitted to an experimental EIC involving 13 scans (*blue*)

After ion tracing and missing value interpolation, we can obtain a list of EICs for each sample. EIC is characterized by its retention time (corresponding to multiple scans), mass value, and ion abundance. In this scenario, the observed data {***t***_*d*_} (same for {***β***_*m*_}) consists of multiple EIC peaks. Each peak is represented by ion abundances across *S* scans with a certain elution profile shape $\mathcal {F}(\cdot)$ as shown in Fig. 2. Using these scan-level features, we model each EIC peak as shown in Eq. (8):

8 $$  t_{d,n}(s) = x_{d,n}\cdot \delta_{d,n}(s)\cdot \mathcal{F}\left(s,\boldsymbol{\phi}_{d,n}\right)+e_{d,n}(s),~s=1,\cdots,S  $$

where, *x*_*d,n*_ is the ion abundance for *n*^th^ compound of *d*^th^ sample; *δ*_*d,n*_(*s*) is a latent indicator to model the missing scans; the chromatographic peak shape is characterized by the exponentially modified Gaussian (EMG) function [17] parameterized by ***ϕ***, as described in Eq. (9), and *e*_*d,n*_(*s*) is the random noise.

9 $$  \begin{aligned} \mathcal{F}\left(s,\boldsymbol{\phi}\right) &= \frac{1}{2}\zeta \exp\left(\frac{1}{2}\zeta\left(2\mu +\zeta \sigma^{2} -2s\right)\right)\\ &\quad\times(1-\text{erf}\left(\frac{\mu+\zeta\sigma^{2}-s}{\sqrt{2}\sigma}\right), ~\boldsymbol{\phi} \doteq \{\mu, \zeta, \sigma\} \end{aligned}  $$

We hypothesize that the data heterogeneity in *t*_*d,n*_ corresponds to the shape of the EIC (characterized by ***ϕ***) as well as ion abundance *x*_*d,n*_.

**Fig. 3:**
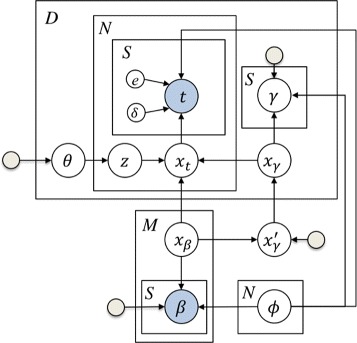
Graphical representation of the scan-level topic model. A lower layer to characterize the scan-level information is added. Ion abundances ***x***_*t*_, ***x***_*β*_, ***x*** *γ*′, and ***x***_*γ*_ together with peak shape (parameterized in ***ϕ***) determined the observed feature list ***t***, ***β***

We extend the purification model we used for integrated peaks by adding a lower layer to characterize the scan-level information as illustrated in Fig. 3. The three assumptions are maintained in this model in terms of the dependancy of ion abundance variables. That is, Eqs. (2)–(7) still hold for ion abundances ***x***_*t*_, ***x***_*β*_, ***x****γ*′, and ***x***_*γ*_. We assume error terms in intensity measurements in Eq. (8) are independent random variables generated by a normal distribution with conjugate prior following an inverse-Gamma distribution:

10 $$ e_{d,n}(s)|\sigma_{e_{d}}^{2} \sim \mathcal{N}\left(0, \sigma_{e_{d}}^{2}\right), ~~~~ \sigma_{e_{d}}^{2} \sim \mathcal{IG}\left(a_{e}, b_{e}\right).  $$

The missing scan indicator variable *δ*_*d,n*_(*s*) follows a Bernoulli distribution, parameterized by *q*_*d*_ with a prior of Beta distribution:

11 $$ \begin{aligned} p\left(\delta_{d,n}(s)|q_{d}\right) &= \text{Bernoulli}\left(\delta_{d,n}(s)|q_{d}\right),~~~~p\left(q_{d}|a_{q},b_{q}\right)\\&=\text{Beta}\left(q_{d}|a_{q},b_{q}\right). \end{aligned}  $$

The observed data point therefore follows the distribution:

12 $$ \begin{aligned} t_{d,n}(s)|{x_{t}}_{d,n},q_{d},\phi_{d,n},\sigma_{e_{d}}^{2}&\sim q_{d}\mathcal{N}\left({x_{t}}_{d,n}\mathcal{F}(s,\phi_{d,n}),\sigma_{e_{d}}^{2}\right)\\&\quad+(1-q_{d})\mathcal{N}\left(0,\sigma_{e_{d}}^{2}\right). \end{aligned}  $$

The peak shape parameters ***ϕ*** are considered to have a normal distribution and its detailed priors are described in [17]. The extended model contains variables that are mutually coupled, providing no analytical form for the posterior distribution in calculation. As a variational approximation, we can split the model into two components: 1) mixture model of underlying ion abundances, and 2) scan-level feature generation. We adopt a two-phase approach to iteratively update the latent variables and estimate the parameters between the two parts. Specifically, we use a Markov chain Monte Carlo (MCMC) sampling method [17] to infer the peak shape model parameters of the second part (i.e., ion abundance ***x***_*t*_, ***x***_*β*_, and shape function parameters *ϕ*). We then treat ***x***_*t*_, ***x***_*β*_ as observed variables to implement the inference on the first part using the same algorithm [8] employed in the intensity-level purification. Once converged, the model outputs the sample-specific mixture proportion ***θ***, pure ion abundance ***x***_*γ*_, shape function parameters ***ϕ*** and related parameters. After purification is performed, ion intensity may be calculated by applying peak detection algorithms [18, 19] to the pure EIC peaks {***γ***_*d,n*_} recovered using Eq. (8).

### Mass spectrometric datasets

The experimental data were acquired by analyses of tissue and blood samples from patients with hepatocellular carcinoma (i.e., HCC, case group) and liver cirrhosis (control group) [1–4]. HCC is a highly heterogeneous disease both at the molecular and clinical levels [20]. Whereas all patients in this study were diagnosed with liver cirrhosis, about half of them were also diagnosed with HCC. Contamination occurs due to the influence from cirrhotic constituents in HCC samples. In this study, we used GC-MS data acquired by analysis of metabolites in 15 tissues and LC-MS data acquired by analysis of proteins in sera from 116 subjects.

#### GC-MS based metabolomic dataset

**Fig. 4:**
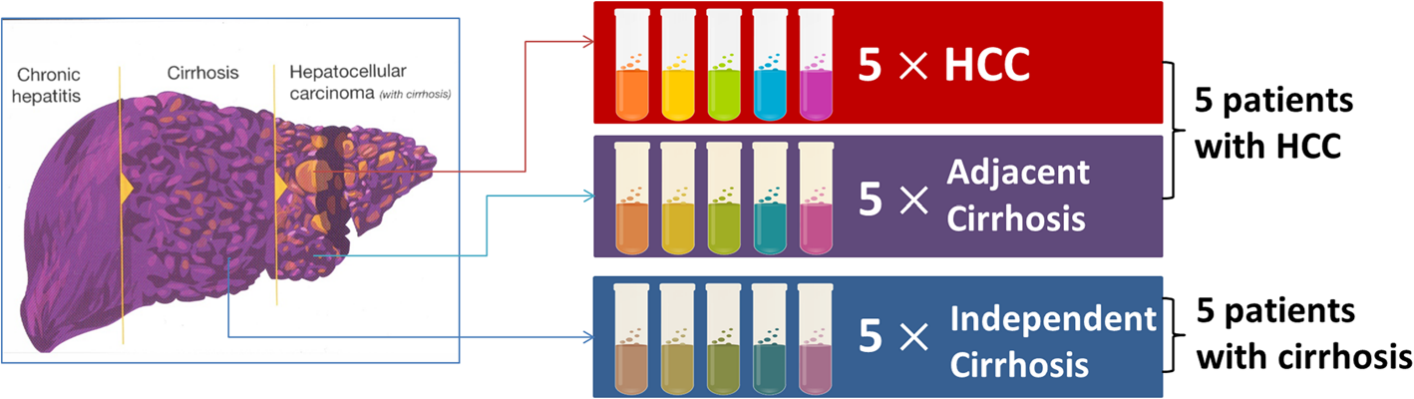
Fifteen tissue samples collected from 10 subjects (5 HCC cases and 5 cirrhotic controls). Five tumor and five adjacent cirrhotic tissues were obtained from the 5 HCC cases. Additional 5 cirrhotic tissues were obtained from the 5 independent subjects with liver cirrhosis

Fifteen liver tissues were collected from 10 participants recruited at MedStar Georgetown University Hospital. As shown in Fig. 4, the tissues were collected from 5 HCC cases (5 tumor and 5 adjacent cirrhotic tissues) and 5 patients with liver cirrhosis. Samples were profiled through Agilent 7890A gas chromatography coupled with LECO’s time-of-flight mass spectrometer to characterize the metabolome alterations associated with HCC development in cirrhotic patients. We identified 559 metabolites after preprocessing the GC-MS raw data by ChromaTOF GC software with True Signal Deconvolution package (Leco Corporation). Two types of purification are investigated on the data. One is to purify HCC profiles by removing contaminants from cirrhotic profiles. The other is to purify adjacent cirrhotic profiles by reducing the impact of the profiles attributed to HCC.

#### LC-MS based proteomic dataset

We acquired 116 proteomic data by analysis of sera from 57 HCC cases and 59 patients with liver cirrhosis recruited from the hepatology clinics at MedStar Georgetown University Hospital. Following depletion and digestion, proteins extracted from sera were injected into a 3000 Ultimate nano-LC system interfaced to LTQ Orbitrap Velos and TSQ Vantage mass spectrometers in untargeted and targeted analyses, respectively. Proteins were identified and quantified by MaxQuant [21] and Skyline [22] in preprocessing untargeted and targeted LC-MS data, respectively. Finally, 101 proteins that were consistently identified across 116 samples were selected as intensity-level features in expression profiles (i.e., *L*=101). All profiles were normalized to the mean total-ion-counts at *N*=1.68×10^8^. It is still not clear how the development of tumor in liver directly affect the alterations in blood. We hypothesize that there are some impacts from cirrhotic constituents contributing to the HCC profile in serum. The contamination may occur in an indirect way through, for example, secreted biomolecules instead of adjacent tissue cells. We apply the purification to remove the influence from cirrhotic contaminants.

#### Synthetic datasets

**Fig. 5:**
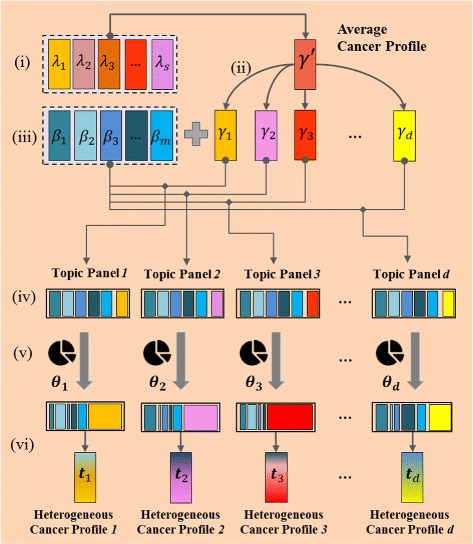
Generative process of heterogeneous cancer profiles. (*i*) average cancer profiles in case group; (*ii*) generate sample-specific pure cancer profile; (*iii*) select sources of contaminants in control group; (*iv*) form topic panels; (*v*) generate sample-specific mixture proportions; (*vi*) generate synthetic cancer profiles

Before applying the models to experimental data, we generated synthetic datasets by artificially mixing real LC-MS data on both intensity and scan levels, and evaluated the model based on its performance of deconvolving the mixed data. We generated synthetic data based on the 116 LC-MS profiled serum proteomic dataset. We assume here that human sera are homogeneous specimens. Hence we can mix them to simulate heterogeneous cancer profiles. Figure 5 shows the generative process of 30 synthetic cancer profiles with contamination, following the steps below: (i) Average the profiles of HCC group, {***λ***_*s*_}_*s*=1,⋯,57_, to obtain an average cancer profile ***γ***^′^, which is close to the real cancerous profile for HCC. (ii) Sample 30 individual pure cancer profiles {***γ***_*d*_}_*d*=1,⋯,30_ from a Dirichlet distribution, as in (4), parameterized by ***γ***^′^ and $\kappa _{d} = \frac {1}{\min _{l}(\gamma '_{l})}$. (iii) Randomly select a subset of cirrhotic profiles {***β***_*m*_}_*m*=1,⋯,*M*_ (*M*=9 in this simulation) as sources of contamination. Normalize them into form of multinomial distribution. (iv) Combine *M* cirrhotic profiles with each of the pure cancer profiles to create 30 topic panels, each consisting of *M*+1=10 profiles. (v) Sample 30 mixture proportions {***θ***_*d*_}_*d*=1,⋯,30_ from a Dirichlet distribution, as in (2), parameterized by ***α***=[1,⋯,1,5], which is uniform for the first nine constituents (contaminants) and with a larger value assigned to last constituent (cancer origin). This ensures a larger proportion of cancerous component in final cancer profile. (vi) Sample a topic indicator *z*_*d,n*_ from ***θ***_*d*_ using (5), and sample a *t*_*d,n*_ from ***β***_*z*_ if *z*≤*M* or ***γ***^***′***^_*d*_ otherwise, as in (6), (7). Repeat the sampling for *N*=1.68×10^8^ times to generate a synthetic cancer profile ***t***_*d*_.

**Fig. 6:**
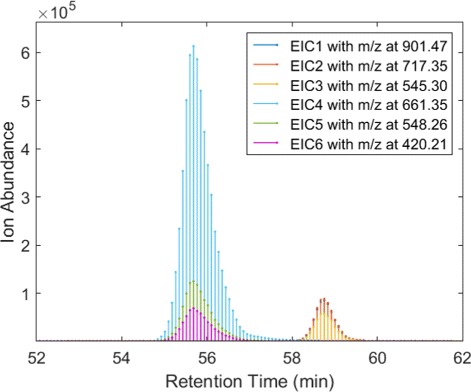
Extracted ion chromatograms from LC-MS based serum proteomic data. Extracted ion chromatogram is characterized by *m*/*z*, retention time, and ion abundance

Each of these 30 heterogeneous cancer profiles is a mixture of a pure cancer profile and multiple contaminants. The intensity-level purification procedure will help retrieve the pure cancer profile and estimate the sample purity as well as proportions of contaminants. Similar to intensity-level simulation, we generated heterogeneous dataset using scan-level features, i.e. EICs, exported from Skyline [22]. Corresponding to 101 proteins, 187 peptides with 561 scan-level features were extracted in each of the 116 samples. Each feature contains 60 scans representing a chromatographic peak as illustrated in Fig. 6. We followed the same steps (i-vi) except that we average and blend EIC peaks instead of protein intensities. Finally, 30 heterogeneous cancerous samples, each characterized by a list of 561 EICs, are generated.

### Evaluation methods

We evaluated the performances of our proposed models on both synthetic and real experimental LC/GC-MS datasets in consideration of the following three goals: 1) to test on intensity level if the model can reasonably estimate the proportion of mixtures in each of the synthetic profiles and recover the pure cancer profiles underneath; 2) to demonstrate if the scan-level purification model gives more accurate estimation on synthetic data; 3) to investigate the benefits of using these models to purify samples from cancer patients collected in our previous differential analysis studies.

Outputs of intensity-level model include the sample-specific mixture proportions $\{\boldsymbol {\theta }_{d}^{*}\}$, pure cancer profiles $\{\boldsymbol {\gamma }_{d}^{*}\}$, and the estimated average cancer profile ***γ***^***′***^^∗^. Whereas, we expect outputs of sample-specific mixture proportion $\{\boldsymbol {\theta }_{d}^{*}\}$, pure ion abundance $\{\boldsymbol {x}_{\gamma }^{*}\}$, peak shape function parameters ***ϕ***^∗^ from extended model. For synthetic datasets, we compare the estimated proportions of mixtures $\{\boldsymbol {\theta }^{*}_{d}\}$ with the true ones ({***θ***_*d*_}) used to generate the synthetic data. Estimation error ratio for a single sample is defined in Eq. (13).

13 $$ \xi_{d}\left(\boldsymbol{\theta}^{*},\boldsymbol{\theta}\right) = \frac{\|\boldsymbol{\theta}^{*}_{d}-\boldsymbol{\theta}_{d}\|_{1}}{\|\boldsymbol{\theta}_{d}\|_{1}}\times 100~\%, ~ d= 1,\cdots, 30  $$

Different from point-wise intensities, the scan-level estimation error ratio for a single sample is defined in Eq. (14)

14 $$ \begin{aligned} \xi_{d}\left(\boldsymbol{\gamma}^{*},\boldsymbol{\gamma}\right) &= \frac{\left\|\sum_{s=1}^{S}\left[\boldsymbol{\gamma}^{*}_{d}(s)-\boldsymbol{\gamma}_{d}(s)\right]\right\|_{1}}{\left\|\sum_{s=1}^{S}\boldsymbol{\gamma}_{d}(s)\right\|_{1}}\\&\quad\times 100~\%, ~ d= 1,\cdots, 30 \end{aligned}  $$

For experimental datasets, we evaluated the performances in multiple aspects including statistical significance of the candidate biomarkers, ROC curves in distinguishing the biological groups, and pathway analysis results.

## Results and discussions

### Synthetic datasets

We applied current model and the extended model to the synthetic intensity-level and scan-level LC-MS datasets, respectively. By incorporating peak detection algorithms, we can further compare the purification performances between the two topic models.

#### Intensity-level purification

**Fig. 7:**
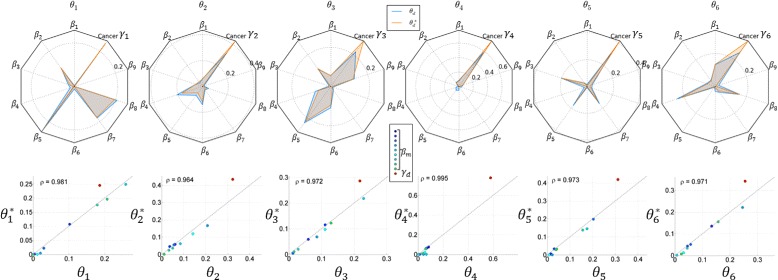
Similarity evaluation on ***θ***. Comparison between estimated ***θ*** ^∗^ and true mixture proportions ***θ*** for the first six profiles. *Top*: radar charts with 10 spokes, each representing a source in topic panel. The proportion of each source is depicted by the length of lines with color (*orange* for estimation ***θ*** ^∗^ and *blue* for ground truth ***θ***). *Bottom*: scatter plots of corresponding proportions in ground truth ***θ*** and estimation ***θ*** ^∗^. The correlation coefficients *ρ* are given on the left-top

**Fig. 8:**
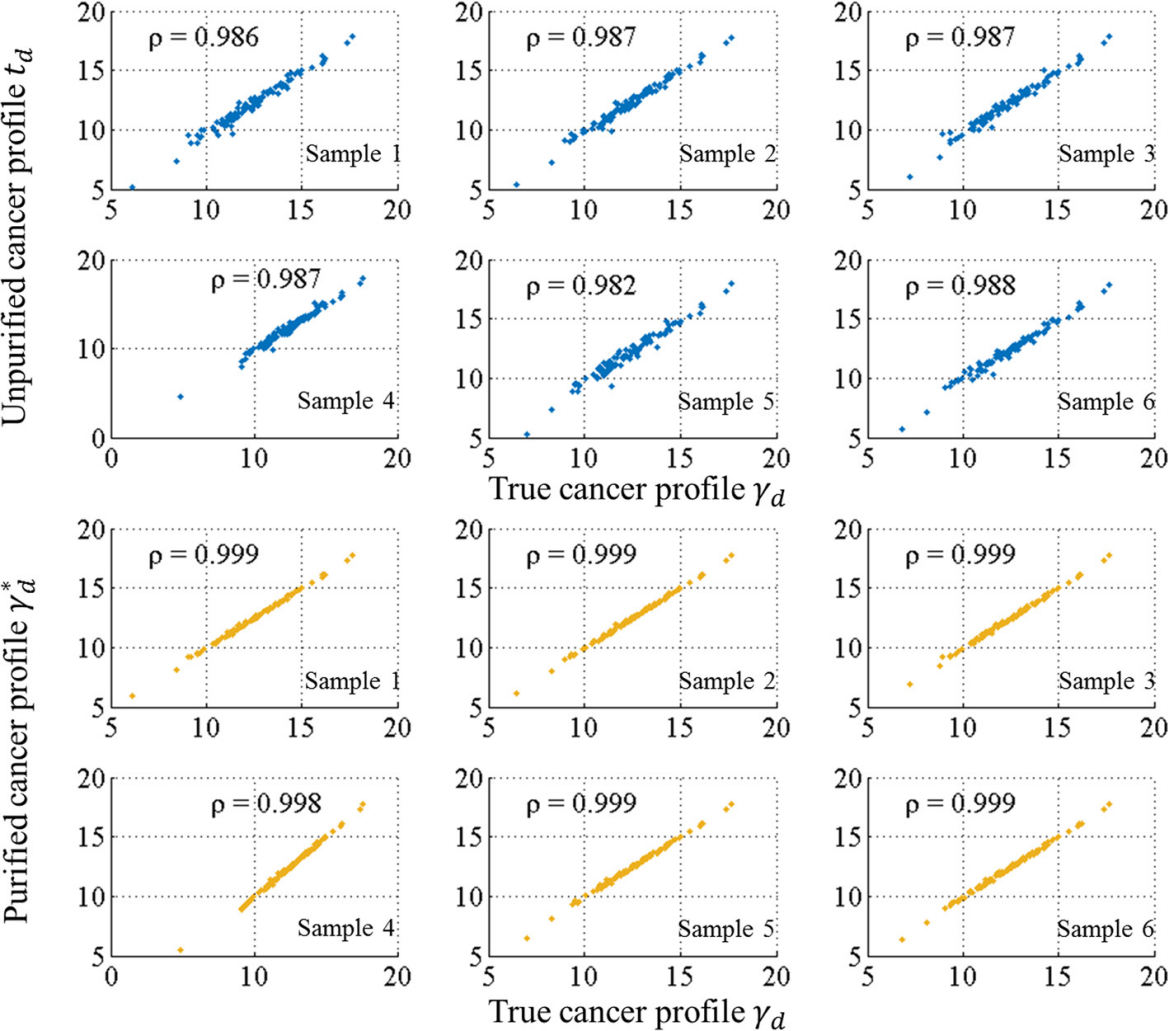
Similarity evaluation on *γ*. The first six out of 30 scatter plots of unpurified cancer profiles versus true cancer profiles (*blue*) and corresponding scatter plots of purified cancer profiles versus true cancer profiles (*orange*). The correlation coefficients *ρ* between each pair of profiles are given on the left-top

**Fig. 9:**
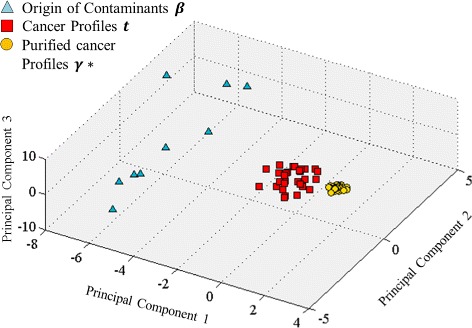
PCA analysis on simulated dataset. Thirty cancer profiles {***t***_*d*_} (*red square*), 30 purified cancer profiles {***γ*** ^***∗***^_*d*_} (*yellow circle*), and 9 sources of cirrhotic contaminants {***β***_*m*_} (*blue triangle*)

We obtained an average error ratio of mixture proportion $\bar {\xi }_{d}(\boldsymbol {\theta }^{*},\boldsymbol {\theta })$ at 2.33 %, indicating a good characterization of original proportions. The comparison of proportion parameters for the first six profiles is depicted in Fig. 7 using radar charts and scatter plots. As shown in the figure, the estimation in each profile has captured consistent patterns as the ground truth in each of the 10 components. We achieved an average correlation coefficient between ***θ***_*d*_ and $\boldsymbol {\theta }^{*}_{d}$ at 0.975. The model accurately recognized those non-cancerous constituents contributed as small as 5 *%* in each sample. The proportion of cancerous origin is overestimated in some samples due to the smaller contributions from the contaminants. The differences between ***θ***_*d*_ and $\boldsymbol {\theta }^{*}_{d}$ are also related to the recovered pure cancer profiles $\{\boldsymbol {\gamma }_{d}^{*}\}$. Similarly, we have the average estimation error ratio for sample-specific pure cancer profiles $\bar {\xi _{d}}(\boldsymbol {\gamma }^{*}, \boldsymbol {\gamma }) = 6.51~\%$, which is smaller than $\bar {\xi _{d}}(\boldsymbol {t}, \boldsymbol {\gamma }) = 16.57~\%$, i.e., the error ratio between unpurified cancer profile and true cancer profile. Figure 8 shows scatter plots of 101 proteins in unpurified cancer profile {***t***_***d***_}_*d*=1,⋯,6_ versus true cancer profile (blue) and in purified cancer profile versus true cancer profile (orange). The average correlation coefficient increases from 0.986 to 0.999 after purification. The effects of purification are illustrated in Fig. 9 by projecting the high-dimensional (dim=101) profiles onto their top three principal components. We observe that the purified cancer profiles were more distant from non-cancerous profiles, and regularized towards an average cancer profile.

#### Scan-level purification

We first evaluated the purification power in the case of scan-level features. The average estimation error ratio of mixture proportions is 3.57 *%* by Eq. (13). In terms of recovering the underneath pure feature list, we achieved the average estimation error ratio for sample-specific pure cancerous feature list $\bar {\xi _{d}}(\boldsymbol {\gamma }^{*}, \boldsymbol {\gamma }) = 3.12~\%$, which is smaller than $\bar {\xi _{d}}(\boldsymbol {t}, \boldsymbol {\gamma }) = 9.61~\%$, i.e., the error ratio between unpurified cancerous feature list and ground truth. The purification with scan-level features works to some extent but it is also interesting to prove the extended model works in a more accurate way than intensity-level topic model. To allow intensity-level purification model to handle scan-level synthetic dataset, we employed peak detection algorithms (i.e., through successive convolution with a 4th order Savitzky-Golay smoothing filter and a first-order derivative of a Gaussian kernel with window width of 25 scans, standard deviation of 3) to transfer EIC peaks into intensities using area under curve. The same algorithm is applied for transferring purified peak list resulted from the extended model. We obtained a greater distance of mixture proportion with $\bar {\xi ^{\mathcal {I}}_{d}}(\boldsymbol {\theta }^{*}, \boldsymbol {\theta })$ at 7.23 *%* if using intensity-level purification model, compared to half ($\bar {\xi ^{\mathcal {S}}_{d}}(\boldsymbol {\theta }^{*}, \boldsymbol {\theta })=3.57~\%$) achieved by extended scan-level purification model.

### LC-MS based proteomic dataset

**Fig. 10:**
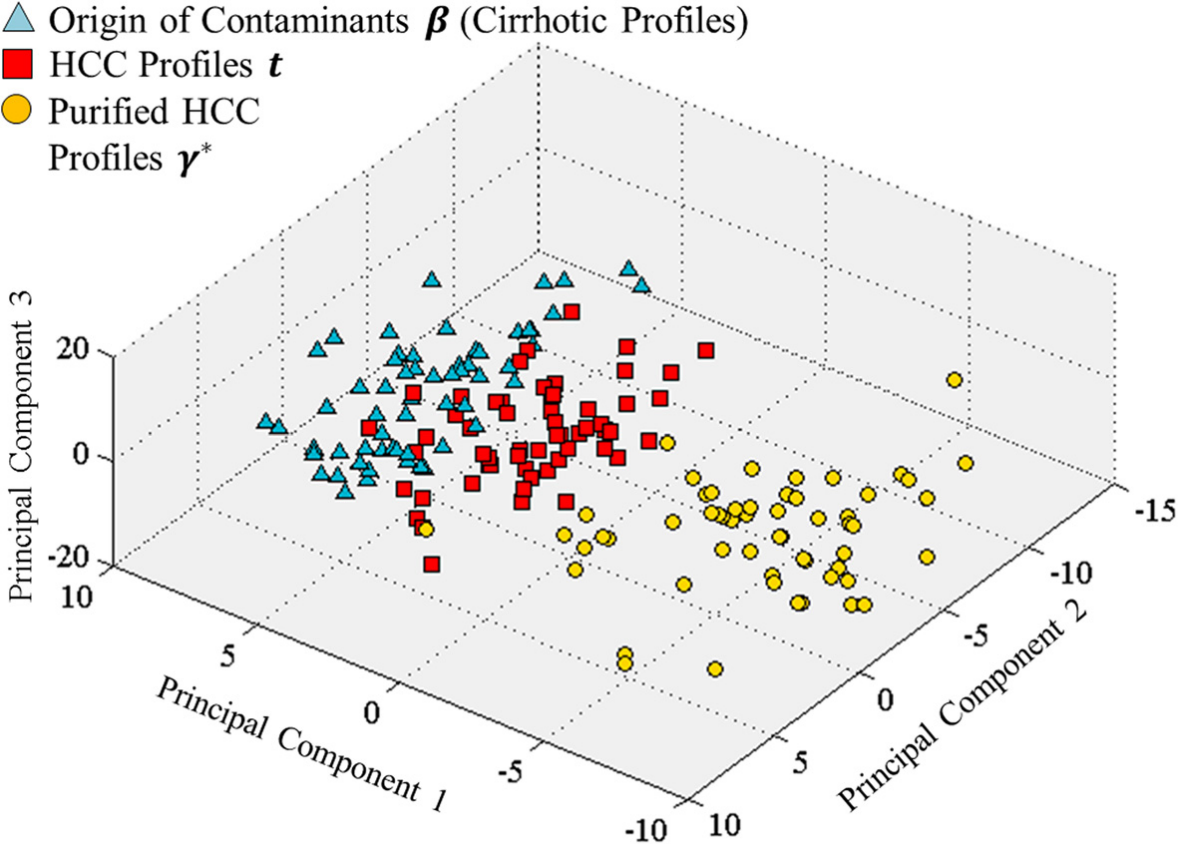
PCA analysis on proteomic dataset. Fifty seven HCC profiles {***t***_*d*_} (*red square*), 57 purified HCC profiles {***γ*** ^***∗***^_*d*_} (*yellow circle*), and 59 sources of cirrhotic contaminants {***β***_*m*_} (*blue triangle*)

We treated all 59 cirrhotic profiles as origins of contaminants to purify 57 HCC profiles. We plotted these profiles using their first three principal components in Fig. 10.

Similar to the simulation result, we observed a clearer distinction between HCC and cirrhotic profiles after purification. To further understand the improvements, we carried out the following analyses on both purified and unpurified profiles.

Firstly, in statistical analysis, the most relevant proteins with differential intensities between HCC cases and cirrhotic controls were selected using t-test, and the associated *p*-values were adjusted based on multiple testing correction (FDR ≤0.05). We found 43 proteins with significant change in expression between the two groups. The number of reported significant proteins under the same testing method increased from 43 to 75 after purification. The majority of the proteins identified in original profiles (40 out of 43) remained significant after purification. If purified based on scan-level features, the number of significant proteins also increased to 69, among which 38 and 61 are overlapped with unpurification and intensity-level purification results, respectively.

**Fig. 11:**
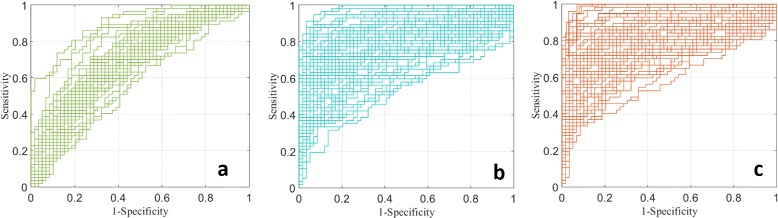
ROC curves of significant proteins. **a** ROC curves for each of 43 significant proteins before purification ($\overline {\text {AUC}} = 0.706, 95~\%~\text {CI}~[0.606, 0.795]$). **b** ROC curves for each of 75 significant proteins after intensity-level purification ($\overline {\text {AUC}} = 0.793, 95~\% \text {CI}~[0.700, 0.863]$). **c** ROC curves for each of 69 significant proteins after scan-level purification ($\overline {\text {AUC}} = 0.811, 95~\%~\text {CI}~[0.719, 0.890]$)

Figure 11a, b, and c show ROC curves for each of the 43, 75, and 69 significant proteins, respectively. A bootstrap method (1000 bootstrap replicates) was used to compute the 95 *%* confidence interval (CI) of the area under each ROC curve. After intensity-level and scan-level purification we respectively achieved an average AUC of 0.793 (with 95 *%* CI at [0.700, 0.863]) and 0.811(with 95 *%* CI at [0.719, 0.890]), both higher than 0.706 (with 95 *%* CI at [0.606, 0.795]) for original biomarkers. More powerful biomarkers were selected after scan-level purification.

Finally, we used DAVID [23] (version 6.7) to identify significant signaling pathways, where the UniProt IDs of the significant proteins were mapped to the KEGG [24] database. As shown in Table 1, three pathways were reported from the original list of significant proteins. Following intensity-level and scan-level purifications, we found peroxisome proliferator-activated receptor (PPAR) signaling pathway with five and six significant proteins involved in addition to the three pathways (complement and coagulation casades, systemic lupus erythematosus, and prion disease) identified without purification. This is interesting in light of previous reports linking cancer and PPARs expressed in human liver [25].

**Table 1:** Signaling Pathways (number of significant proteins involved in the pathway)

Without purification	Intensity-level purification	Scan-level purification
Complement and coagulation cascades (13)	Complement and coagulation cascades (18)	Complement and coagulation cascades (19)
Systemic lupus erythematosus (5)	Systemic lupus erythematosus (6)	PPAR signaling pathway (6)
Prion diseases (4)	PPAR signaling pathway (5)	Systemic lupus erythematosus (4)
-	Prion diseases (4)	Prion diseases (4)

### GC-MS based metabolomic dataset

Heterogeneity issue is more intuitive in tissue samples, where the contaminations originate from the neighboring non-homogeneous cells. We first purified the HCC profiles {***t***_*d*_}_*d*=1,⋯,5_ using independent cirrhotic profiles {***β***_*m*_}_*m*=1,⋯,5_ as the sources of contamination. Without purification, none of the 559 metabolites passed the statistical test as significant (FDR adjusted *p*-value ≤0.05). However, seven metabolites were identified as significant after the profiles were purified. For the adjacent cirrhotic profiles {***ψ***_***d***_}_*d*=1,⋯,5_, we applied the model to remove contaminations from any neighboring cancerous cells. We expected to observe that the purified adjacent cirrhotic profiles became close to independent cirrhotic profiles. The dissimilarity, defined in (8), between independent and adjacent cirrhotic profiles is $\bar {\xi }(\boldsymbol {\psi },\boldsymbol {\beta }) = 28.3~\%$, and goes down to $\bar {\xi }\left (\boldsymbol {\psi ^{*}},\boldsymbol {\beta }\right) = 24.9~\%$ after purification. The improvements are less substantial compared to the previous datasets, presumably due to the limited sample size and potential overfitting issue.

## Conclusions

In this paper, we investigate topic model-based inference methods to computationally address heterogeneity issue in samples analyzed by LC/GC-MS. The topic model gives a probabilistic explanation on the corpus of LC/GC-MS based profiles on both integrated peak and scan-level ion intensity levels. The performances of our models in estimating mixture proportion and retrieving underlying true cancer profile are evaluated through well-designed synthetic data. We observed that incorporation of scan-level features gives more accurate purification results by alleviating the loss in information caused as a result of integrating peak intensity values. Through GC-MS metabolomic and LC-MS proteomic datasets we acquired from tissues and blood samples, respectively, we showed the benefit of applying topic-model based purification of the data prior to statistical and pathway analyses. Specifically, we observed improved discrimination between case and control groups and biologically meaningful pathway analysis results. Future studies will focus on cross-validation of the findings either computationally through mass spectrometric data from large-scale cancer biomarker discovery studies or by using ground-truth information from pathology reports and literature survey.

## Abbreviations

EIC, extracted ion chromatogram; GC, gas chromatography; HCC, hepatocellular carcinoma; LC, liquid chromatography; MS, mass spectrum; RT, retention time
